# Prevalence and Diversity of *Salmonella* Serotypes in Ecuadorian Broilers at Slaughter Age

**DOI:** 10.1371/journal.pone.0159567

**Published:** 2016-07-14

**Authors:** Christian Vinueza-Burgos, María Cevallos, Lenin Ron-Garrido, Sophie Bertrand, Lieven De Zutter

**Affiliations:** 1 Facultad de Medicina Veterinaria y Zootecnia, Universidad Central del Ecuador, Quito, Ecuador; 2 National Reference Centre for *Salmonella* and *Shigella*, Bacterial Diseases Division, Communicable and Infectious Diseases, Scientific Institute of Public Health (WIV-ISP), Brussels, Belgium; 3 Department of Veterinary Public Health and Food Safety, Faculty of Veterinary, Ghent University, Merelbeke, Belgium; 4 Centro Internacional de Zoonosis, Quito, Ecuador; Institut National de la Recherche Agronomique, FRANCE

## Abstract

*Salmonella* is frequently found in poultry and represent an important source for human gastrointestinal infections worldwide. The aim of this study was to investigate the prevalence, genotypes and antimicrobial resistance of *Salmonella* serotypes in broilers from Ecuador. Caeca content from 388 at random selected broiler batches were collected in 6 slaughterhouses during 1 year and analyzed by the ISO 6579/Amd1 protocol for the isolation for *Salmonella*. Isolates were serotyped and genotypic variation was acceded by pulsed field gel electrophoresis. MIC values for sulfamethoxazole, gentamicin, ciprofloxacin, ampicillin, cefotaxime, ceftazidime, tetracycline, streptomycin, trimethropim, chloramphenicol, colistin, florfenicol, kanamycin and nalidixic acid were obtained. Presence of *bla*_CTX-M_, *bla*_TEM,_ *bla*_SHV_ and *bla*_CMY_; and *mcr-1* plasmid genes was investigated in resistant strains to cefotaxime and colistin respectively. Prevalence at batch level was 16.0%. The most common serotype was *S*. Infantis (83.9%) followed by *S*. Enteritidis (14.5%) and *S*. Corvallis (1.6%). The pulsed field gel electrophoresis analysis showed that *S*. Corvallis, *S*. Enteritidis and *S*. Infantis isolates belonged to 1, 2 and 12 genotypes respectively. *S*. Infantis isolates showed high resistance rates to 12 antibiotics ranging from 57.7% (kanamycin) up to 98.1% (nalidixic acid and sulfamethoxazole). All *S*. Enteritidis isolates showed resistance to colistin. High multiresistant patterns were found for all the serotypes. The *bla*_CTX-M_ gene was present in 33 *S*. Infantis isolates while *mcr-1* was negative in 10 colistin resistant isolates. This study provides the first set of scientific data on prevalence and multidrug-resistant *Salmonella* coming from commercial poultry in Ecuador.

## Introduction

Foodborne infections in humans caused by *Salmonella* are of primary importance around the world. Majowicz et al. [1] estimated that non-typhoidal *Salmonella* was the cause of 93.8 million cases of gastroenteritis, with 155.000 deaths yearly worldwide. For 2010 it was estimated that foodborne salmonellosis caused by non-typhoidal *Salmonella* resulted in 6.43 million Disablility-Adjusted Live Years [2]. Salmonellosis is characterized by acute onset of fever, abdominal pain, diarrhea and nausea [3]. Salmonellosis is especially important in susceptible groups such as young, elderly and immunocompromised patients [2]. In Ecuador 3373 human cases or 21.6 cases/100,000 inhabitants of foodborne salmonellosis were reported in 2014 [4].

Although *Salmonella* contaminated vegetables and fruits may be the source for human infection, several domestic animal species are considered as the most important source of human infection, since such animals are often colonized by this pathogen. Poultry is by far the main vehicle of these pathogens in the food chain [5–7]. In Latin America some *Salmonella* outbreaks in humans linked to chicken consumption are published [8–10]. However data on the prevalence of *Salmonella* in industrial reared poultry in Latin America is limited [11–13].

Worldwide the use of antibiotics in husbandry practices is a major concern since this may promote the development of multidrug-resistant bacteria. Antibiotics in poultry production systems are widely used to prevent, control and treat bacterial infections as well as growth promoters [14]. These facts are of special relevance in developing countries where misuse of antibiotics and the lack of control over their usage is a problem to be addressed [15]. Resistant bacteria can cause human diseases or transmit its resistance genes to pathogenic bacteria [16].

In Ecuador chicken meat is frequently consumed and its demand increased over the years [17]. Although Ecuadorian poultry industry only provides chicken meat for local consumption, it is expected that in the future it can have access to international markets once sanitary conditions are better understood and controlled. Moreover, despite the importance of non-typhoidal *Salmonella* as a foodborne pathogen, little is known about its epidemiology on poultry farms, in slaughterhouses and retail stores in the main centers of production and consumption of poultry products. This Information may help to establish surveillance programs and interventions measures regarding the presence and antimicrobial resistance of *Salmonella*.

The aim of this study was to investigate the prevalence, genetic profiles and antimicrobial resistance of *Salmonella* in broilers slaughtered in industrial facilities located in the province of Pichincha in Ecuador.

## Materials and Methods

### Study design and sampling

Pichincha, the province where Quito the capital city of Ecuador is located, was selected as the area to collect samples since it is an important region within Ecuador for the production of broiler meat. Big slaughterhouses were contacted and asked for their willingness to cooperate in the study. Based on these results sampling was performed in 6 slaughterhouses. From June 2013 to July 2014, a total of 388 batches (birds coming from one broiler house and slaughtered on the same day) were sampled. Each batch originated from a different epidemiological unit. All sampled batches were commercially reared and slaughtered at the age of 6 to 7 weeks.

From each batch one caecum from 25 randomly selected chickens were collected, and transported in an ice box within 1 hour to the laboratory for bacteriological analysis.

### Isolation and Identification of *Salmonella*

From each of the 25 caeca content was aseptically pooled. Therefore, all caeca were immersed in ethanol, and after evaporation of the ethanol approximately 1 g content/cecum was collected in a sterile plastic bag. All samples were homogenized by hand during 1 min. after the addition of 225 ml Buffered Peptone Water (BPW; Difco, BD, Sparks, MD). After the incubation of the preenrichment media at 37°C for 20 hours 3 drops of each culture medium were spotted onto a Modified Rappaport-Vassiliadis agar plate (MSRV; Oxoid, Basingstoke, UK) and incubated at 42°C for 24 hours. Plates were examined for migration and if present a loopful from the edge of the migration zone was streaked onto a Xylose Lysine Deoxycholate agar plate (XLD, Difco) and incubated at 37°C for 24 hours. Two presumptive *Salmonella* colonies were tested using Triple Sugar Iron agar (Difco, BD), Lysine Iron agar (BBL, BD), Urea agar (BBL, BD) and Sulfur Indole Motility medium (BBL, BD) for confirmation.

### Characterization of *Salmonella* isolates

One *Salmonella* isolate per positive sample was further characterized. To limit the number of *Salmonella* strains to be serotyped, isolates were grouped by an enterobacterial repetitive intergenic consensus (ERIC) PCR as described by Rasschaert et al. [18]. ERIC PCR was performed on 59 strains within the same run. Based on ERIC PCR profiles 16 isolates were selected for serotyping. All these selected isolates and the 3 isolates not included in the ERIC PCR run were serotyped according to the Kauffmann-White scheme.

To characterize the *Salmonella* strains within each serotype, all isolates were genotyped by pulse field gel electrophoresis (PFGE) after digestion with *XbaI* enzyme [19]. The relatedness among the PFGE profiles was analyzed with GelCompar II software v. 6.6 (Applied Maths, Sint-Martems-Latem, Belgium). Bands representing fragments between 35 kb and 1140 kb in size were included in the analysis. A similarity dendrogram was constructed by the unweighted pair group method using arithmetic averages algorithm (UPGMA). DICE similarity coefficient with a position tolerance of 1.4 was calculated. A PFGE genotype was assigned on the basis of the difference in the presence of at least one band in the *XbaI* fingerprint [20]. Genotypes were identified by numerical suffixes after a capital indicating the serotype (e.g. I-1 refers to serotype Infantis).

### Antimicrobial Resistance

Antimicrobial resistance was evaluated by determining the minimum inhibitory concentration (MIC) using the EUMVS2 plates (Thermo Scientific, West Palm Beach, USA). The tests were performed according to the manufacturer instructions. The following antibiotics were evaluated: sulfamethoxazole, gentamicin, ciprofloxacin, nalidixic acid, ampicillin, cefotaxime, ceftazidime, tetracycline, streptomycin, trimethoprim, chloramphenicol, colistin, florfenicol and kanamycin. *Escherichia coli* ATCC 25922 was used as the quality control strain. Clinial breakpoints values from the Clinical and Laboratory Standards Institute [21] were considered to determine bacterial antibiotic resistance for kanamycin and sulfamethoxazole. For all other antibiotics epidemiological breakpoint values from the European Committee on Antimicrobial Susceptibility Testing were considered [22]. *Salmonella* isolates resistant to cefotaxime where further examined for the presence of ESBL or AmpC phenotypes by disk diffusion tests [23,24]. According to the disk diffusion results PCR tests were performed to identify *bla*_CTX-M_, *bla*_TEM_ and *bla*_SHV_ genes in ESBL isolates and *bla*_CMY_ in AmpC isolates. PCR conditions and primers were the ones described by Hasman et al. [25] for *bla*_CTX-M_, Olesen et al. [26] for *bla*_TEM_, Arlet et al. [27] for *bla*_SHV_ and; Hasman et al. [25] and Kruger et al. [28] for *bla*_*CMY*_

Isolates with phenotypic resistance to colistin were tested for the presence of the new described *mcr-1* plasmid gene by primers described by Liu et al. [29]. For the PCR reaction mixture the Maxima Hot Start Green PCR Master Mix (Promega) was used. The total mixture of 25 μl contained 1 X hot start PCR buffer, 400μM of each nucleotide (dNTP) 4mM MgCl_2_, 0.2 μM of each primer and 1 μl of the template DNA obtained after boiling during 10 minutes of 1 colony of the bacteria in 100 μl of DNA free water. The following PCR program was used: a denaturation step at 95°C for 5 minutes, 35 cycles of 1 minute at 95°C, 0.5 minutes at 60°C, 1 minute at 72°C, and finally 10 minutes at 72°C. After the PCR, the amplification products were confirmed by gel electrophoresis using a 2% agarose gel. A PCR amplicon of 308 bp was expected. As positive control we used the *Salmonella* autoagglutinable strain S15FP06306, a strain isolated from poultry and confirmed to have the *mcr-1* gene by sequencing of the PCR product and by performing whole genome sequencing on the strain.

### Statistical analysis

Prevalence of *Salmonella* positive batches was estimated using a random-effects logistic regression model with farms and the sampling occasions per farm as random factors. The 95% confidence interval (CI_95%_) for the prevalence was calculated once the regression model fit the intercept. Variance components and their standard deviations and the intraclass correlation coefficient (ICC) are reported. Function *glmer* from *lme4* package [30] in R environment version 3.3.1 [31] was used to estimate the fixed and the random factors. *Salmonella* prevalence in farms and its CI_95%_ were estimated under independence assumption for farms and considering a farm positive when at least one of the sampled batches was positive.

## Results

In total 388 batches originated from 119 farms (1 to 9 flocks per farm) were sampled. From all tested batches 62 (16.0%; CI_95%_: 12.6–24.5) were *Salmonella* positive. The variance component for farms was 0.0237 (SD: 0.154) and 0.0345 (SD: 0.185) for sampling occasions per farm. Thus, the ICC estimated was 0.5928 as a measure of reproducibility in the sample results. Positive batches originated from 50 (42.0%; CI_95%_: 33.1–51.4) farms (Table 1). For 87 farms, more than one batch was sampled. One, two and three batches were found *Salmonella* positive on 41, 6 and 3 of those farms respectively.

**Table 1:** *Salmonella* positive batches in relation to the number of tested batches per farm.

Number of batches/farm sampled	Number of farms	Number of farms with 0, 1, 2 or 3 positive batches			
		0	1	2	3
1	34	27	7		
2	18	12	6		
3	12	7	5		
4	19	10	8	1	
5	17	5	10		2
6	15	6	4	4	1
7	2	1		1	
8	1		1		
9	1	1			
Total	119	70	41	6	3

ERIC-PCR of the 59 *Salmonella* isolates delivered 2 patterns. Serotyping demonstrated that pattern 1 corresponded to *S*. Enteritidis and pattern 2 to *S*. Infantis (Fig A in S1 File). Direct serotyping of the other 3 *Salmonella* strains resulted in 2 strains belonging to *S*. Infantis and 1 strain to *S*. Corvallis. In total 52 isolates (83.9%) were *S*. Infantis, 9 (14.5%) *S*. Enteritidis and 1 (1.6%) *S*. Corvallis.

The PFGE analysis (Fig B in S1 File) showed that *S*. Corvallis, *S*. Enteritidis and *S*. Infantis isolates belonged to 1, 2 and 12 genotypes respectively (Table 2).

**Table 2:** *Salmonella* genotypes present in each serotype.

Serotype	Genotype	Nb. of strains
S. Corvallis	C-1	1
S. Enteritidis	E-1	5
	E-2	4
S. Infantis	I-1	21
	I-2	6
	I-3	2
	I-4	6
	I-5	1
	I-6	1
	I-7	1
	I-8	10
	I-9	1
	I-10	1
	I-11	1
	I-12	1
Total		62

Within the *S*. Infantis strains the genetic similarity was minimal (87% similarity) and the different genotypes were due to the presence or absence of one band in the obtained profiles. The genotype I-1 was the dominant genotype (40.4%) within this serotype. *Salmonella* isolates from 9 farms with more than 1 *Salmonella* positive batch, belonged to different serotypes (2 farms), genotypes (5 farms) or serotypes and genotypes (1 farm) (Table 3).

**Table 3:** *Salmonella* serotypes and genotypes found in farms with multiple positive batches.

Farm	Serotypes-genotypes									Total
	C-1	E-1	E-2	I-1	I-2	I-8	I-9	I-10	I-11	
A							1		1	2
B				1		1				2
C				2						2
D		1	1							2
E		1						1		2
F	1				1					2
G				1	2					3
H				1	2					3
I			1	1			1			3

Antimicrobial resistance rates within each *Salmonella* serotype against the 14 tested antibiotics are shown in Table 4 and the MIC distributions for the different antibiotics are shown in Table B in S1 File. *S*. Infantis isolates showed a resistance rate of 5.8% and 1.9% for ceftazidime and colistin respectively, whereas for the other 12 tested antibiotics the resistance rates varied from 57.7% (kanamycin) up to 98.1% (nalidixic acid and sulfamethoxazole). In contrast, all *S*. Enteritidis isolates showed resistance to colistin. The resistance rate for the other antibiotics ranged from 11.1% up to 33.3%.

**Table 4:** Number of *Salmonella* strains resistant to each tested antibiotic.

	Number (%) of resistant isolates		
Antibiotic	*S*. Infantis	*S*. Enteritidis	*S*. Corvallis
Sulfamethoxazole	51 (98.1)	3 (33.3)	1 (100)
Nalidixic acid	51 (98.1)	2 (22.2)	
Ciprofloxacin	49 (94.2)	2 (22.2)	1 (100)
Tetracycline	49 (94.2)	1 (11.1)	
Trimethropim	47 (90.4)	2 (22.2)	1 (100)
Streptomycin	47 (90.4)	2 (22.2)	
Cefotaxime	42 (80.8)	2 (22.2)	
Ampicillin	41 (78.8)	1 (11.1)	
Florfenicol	40 (76.9)	2 (22.2)	
Gentamicin	39 (75)	2 (22.2)	
Chloramphenicol	39 (75)	1 (11.1)	
Kanamycin	30 (57.7)	2 (22.2)	
Colistin	1 (1.9)	9 (100)	
Ceftazidime	3 (5.8)	1 (11.1)	

*S*. Infantis isolates showed 19 resistance patterns in which resistance from 2 up to 13 antibiotics were involved (Table 5). The resistance pattern 2 (38.5%) was the most frequent one within *S*. Infantis isolates. *S*. Enteritidis isolates presented 4 antibiotic resistance patterns containing 1 (pattern 24, 6 strains), 2 (pattern 21, 1 strain) and 12 (patterns 4 and 5, both one strain) antibiotics. Two *S*. Enteritidis isolates were resistant to 12 antibiotics. The *S*. Corvallis isolate was resistant to 3 antibiotics.

**Table 5:** Antibiotic resistance patterns of *Salmonella* strains and phenotypes of cefotaxime resistant strains.

Pattern	Resistance pattern	No. Antibiotics	*S*. Infantis	*S*. Enteritidis	*S*. Corvallis	Rate (%)	ESBL + strains*	*bla*_CTX-M_	AmpC + strains*
1	SGCAFZTRMHNKL	13	2			3.2%	2	2	
2	SGCAFTRMHNKL	12	20			32.3%	15	15	5
3	SGCAFTRMHONL	12	1			1.6%			1
4	SGCAFTRMONKL	12		1		1.6%			1
5	SGCFZRMHONKL	12		1		1.6%			1
6	SGCAFTRMHNL	11	6			9.7%	6	5	
7	SGCAFTMHNKL	11	2			3.2%	2	2	
8	SGAFTRMHNL	10	1			1.6%	1	1	
9	SGCAFTRMKL	10	1			1.6%	1	1	
10	SGCAFTRHNL	10	1			1.6%	1	1	
11	SGCAFMHNKL	10	1			1.6%	1	1	
12	SGCTRMHNKL	10	3			4.8%	NA	NA	NA
13	SCAFTRMNL	9	1			1.6%			1
14	GCAFMHNKL	9	1			1.6%	1	1	
15	SCTRMHNL	8	1			1.6%	NA	NA	NA
16	SCAFZTRL	8	1			1.6%	1	1	
17	SCAFTRL	7	3			4.8%	3	3	
18	SCFTRML	7	1			1.6%			1
19	SCTRML	6	4			6.5%	NA	NA	NA
20	STRML	5	1			1.6%	NA		NA
21	SCM	3			1	1.6%	NA		NA
22	SO	2		1		1.6%	NA		NA
23	SM	2	1			1.6%	NA		NA
24	O	1		6		9.7%	NA		NA
Total			52	9	1		34	33	10

Sulfamethoxazole (S), ciprofloxacin (C), nalidixic acid (L), tetracycline (T), trimethoprim (M), cefotaxime (F), ampicillin (A), florfenicol (N), gentamicin (G), chloramphenicol (H), kanamycin (K), streptomycin (R), colistin (O) and ceftazidime (Z).

NA: Not Applicable.

*Number of strains with ESBL or AmpC phenotype according to disk diffusion test.

From the 44 *Salmonella* isolates that showed resistance to cefotaxime 34 presented a ESBL phenotype and were *S*. Infantis, while 10 presented an AmpC phenotype with 2 *S*. Enteritidis and 8 *S*. Infantis. None of the ESBL isolates were positive by PCR for the *bla*_TEM_ or *bla*_SHV_ genes, while 33 of these isolates were positive for the *bla*_CTX-M_ gene. None of the AmpC isolates were positive for the *bla*_CMY_ gene. None of the 10 colistin resistant strains were positive for the *mcr-1* plasmid gene by PCR.

## Discussion

To our knowledge, this is the first study about *Salmonella* in commercial reared broiler batches at slaughter in Ecuador. Results indicate that 15.9% of the batches slaughtered in the province of Pichincha are *Salmonella* positive. This result is similar to the prevalence reported in Venezuela (23%; n = 332) [32]. In contrast prevalence in Brazil was only of 5% (n = 40) [33] and in Colombia 65% (n = 315) [34]. On the other hand, for the European Union member states and 3 European non-member states an overall *Salmonella* prevalence of 3.37% at farm level was reported with rates varying from 0.08% in Norway to 13.48% in Hungary in 2014 [35].

Only *S*. Infantis (83.9%), *S*. Enteritidis (14.5%) and *S*. Corvallis (1.6%) were found in positive batches. These findings contrast with data from Colombia, where a wider diversity of *Salmonella* serotypes were reported in broilers at slaughter age [36]. These authors found 31 serotypes among 378 examined *Salmonella* strains with the most common serotypes being *S*. Paratyphi B dT+, *S*. Heidelberg, *S*. Enteritidis and *S*. Typhimurium. Similarly, data from Venezuela indicated that the most prevalent *Salmonella* serotypes at slaughterhouse level were *S*. Parathyphi B and *S*. Heidelberg [32]. On the other hand, in Brazil the most prevalent serotypes in chicken carcasses were *S*. Enteritidis, *S*. Infantis, *S*. Typhimurium and *S*. Heidelberg [37]. In the European Union the most reported serotypes at farm level were *S*. Infantis (43.4%) followed by *S*. Mbandaka (13.5%), *S*. Livingstone (7.3%) and *S*. Enteritidis (7.3%) in 2014 [35]. Accordantly, the emergence of *S*. Infantis in human salmonellosis has been reported [38]. The role poultry in human salmonellosis caused by *S*. Infantis in Ecuador needs further research.

Moreover, PFGE analysis demonstrated that the *S*. Infantis strains were genetically very similar. Although there were 12 identified genotypes within *S*. Infantis, most of them varied in 1 to 2 bands with similarities above 88%, which suggest that these strains are highly related [20]. This is in accordance with other studies that showing a high similarity of *S*. Infantis within poultry, other animal and human isolates [39–42].

The reason why only 3 *Salmonella* serotypes were found and the *S*. Infantis strains showed a high genetic similarity in the present study is not clear and need further research for clarification. In a first step collection of samples from all over Ecuador may give a broader view of *Salmonella* serotypes present in broilers at national level. Moreover, such a study may also confirm the prevalence of *Salmonella* in broilers observed in the present study.

High antibiotic resistance rates were shown against most of the tested antibiotics within *S*. Infantis strains. *S*. Infantis strains showed also higher multiresistant patterns than *S*. Enteritidis. Of the *S*. Infantis strains 44.2% showed resistance to at least 12 antibiotics, whereas 22.2% of *S*. Enteritidis strains presented resistant patterns to 12 antibiotics. In concordance, for Brazil 71.3% (n = 87) of *Salmonella* strains isolated from poultry houses were reported to be resistant to chloramphenicol, ampicillin, ceftazidime, ciprofloxacin, nalidixic acid, tetracycline, sulfamethoxazole, and trimethoprim/sulfamethoxazole [43]. Although *S*. Enteritidis has been found to be susceptible to most antibiotics [44,45], antibiotic resistance has also been reported to β-lactam antibiotics, sulfonamides, quinoxalines, fluoroquinolones and tetracyclines [46–48]. Moreover, 2 *S*. Enteritidis isolates presented resistance towards 12 antibiotics which is in accordance with previous findings [49]. This is of special interest since it suggests that in high antibiotic pressure environments, non-classical multidrug resistant (MDR) *Salmonella* serotypes can emerge.

In the present study 85.5% and 83.9% of *Salmonella* strains were resistant to nalidixic acid and ciprofloxacin respectively. High resistance rates to fluoroquinolones have been reported in *Salmonella*. For example, EFSA and ECDC reported for 2013 high to extremely high levels of resistance to these 2 antibiotics in *Salmonella* from broilers [45]. A study in Serbia showed that 100% of *S*. Infantis strains were resistant to ciprofloxacin and nalidixic acid [42] while Rahmani et al., demonstrated high fluoroquinolone resistance in both, *S*. Infantis and *S*. Enteritidis [41]. High fluoroquinolone resistance rates reported in our study may be explained by the selective pressure of resistant strains under the common use of fluoroquinolones as therapeutics in Ecuadorian broiler farms.

Low rates of colistin resistant in *Salmonella* has been described before [41,50,51]. However, it has been suggested that *S*. Enteritidis may have increased colistin MIC values [52]. This is in accordance with our results where 77.8% of *S*. Enteritidis and 1.9% of *S*. Infantis strains presented a colistin resistant phenotype. On the other hand, other studies have reported that resistance to colistin in *Salmonella enterica* isolated from food animals was mainly presented in *S*. Typhimurium but not in *S*. Enteritidis or S. Infantis [53,54]. Since the resistance in the phenotype positive *Salmonella* strains was not attributable to the *mcr-1* plasmid gene, it may be assumed that mutations in the chromosomal genes were the source for the observed resistance [29]. Even though the *mcr-1* plasmid gene has been mainly described in *E*. *coli* from Latin America, Europe and Asia [29,55–57] this gene has also been observed in *Salmonella enterica* from European countries like UK, Spain and France [58–60]. These data suggest that *mcr-1* gene might be present in *Salmonella enterica* in Latin America, but further research is needed to confirm this assumption.

In accordance with findings from other studies carried out in Latin America, β-lactam-resistant *Salmonella* isolates were identified [34,61,62]. Although *bla*_TEM_ and *bla*_SHV_ are reported as common genes in resistant *Salmonella* [43,63], these resistance genes were not found in our strains. However, studies in Brazil and USA have identified the *bla*_*CTX-M*_ genes as the most prevalent ESBL genes in *Salmonella* recovered from poultry [64,65] which is in accordance with our results. It should be taken into account that, even though the main families of beta-lactamases were included in this study, resistance to beta-lactams present in the negative strains could be mediated by other ESBL or AmpC genes [14,66]. The presence of these strains in Ecuadorian broilers is of public health concern since resistance to β-lactam antibiotics, listed as WHO Essential Medicines [67], may limit the options to treat human *Salmonella* infections.

Moreover, all antibiotics, with exception of colistin and ceftazidime, showed high rates of antimicrobial resistance indicating the necessity of a better use of antibiotics and biosecurity implementation in the primary sector to reduce the multidrug-resistant bacteria loads in broilers reared in Ecuador. It is worth to mention that there is a global trend towards an increase of antimicrobials consumption in the animal production sector [68]. This place a concern since the misuse of antibiotics in livestock production can lead to the occurrence of MDR bacteria, especially in low- and middle-income countries frequently lacking a clear legislative framework about the use of antibiotics in the animal production sector [69].

In conclusion, this study provides the first set of scientific data on prevalence and multidrug-resistant *Salmonella* originating from commercial poultry in Ecuador. This evidence may be useful for implementation of official policies aiming to decrease the prevalence of *Salmonella* in poultry farms.

## Supporting Information

S1 File Fig A, ERIC-PCR profiles of the 59 tested *Salmonella* isolates. Fig B, PFGE profiles of the 62 *Salmonella* isolates collected from the positive broiler batches. Table A, Distribution of the minimal inhibitory concentration values for the 62 *Salmonella* isolates collected from the positive broiler batches.
(PDF)
