# Supplementary material for: Prevalence and Diversity of Salmonella Serotypes in Ecuadorian Broilers at Slaughter Age
Source: PLoS One. 2016 Jul 14;11(7):e0159567. doi: 10.1371/journal.pone.0159567 (PMC4944992; doi:10.1371/journal.pone.0159567)
Supplement: S1 File — Fig A, ERIC-PCR profiles of the 59 tested Salmonella isolates. Fig B, PFGE profiles of the 62 Salmonella isolates collected from the positive broiler batches. Table A, Distribution of the minimal inhibitory concentration values for the 62 Salmonella isolates collected from the positive broiler batches. (PDF) [file pone.0159567.s001.pdf]

Figure A. ERIC-PCR profiles of the 59 tested *Salmonella* isolates.

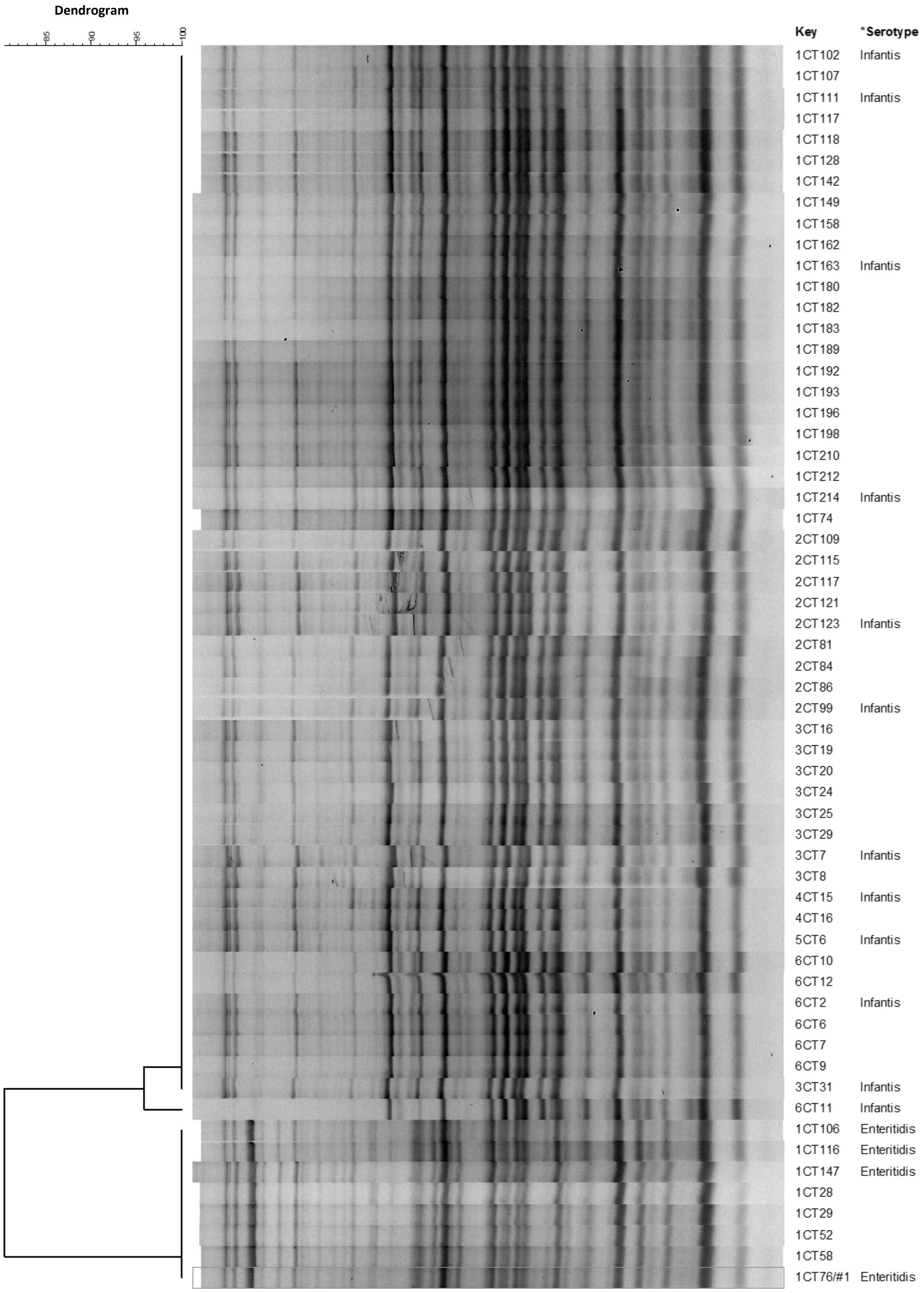

* Selected isolates for serotyping according to the Kaufmann-White scheme

Figure B. PFGE profiles of the 62 *Salmonella* isolates collected from the positive broiler batches.

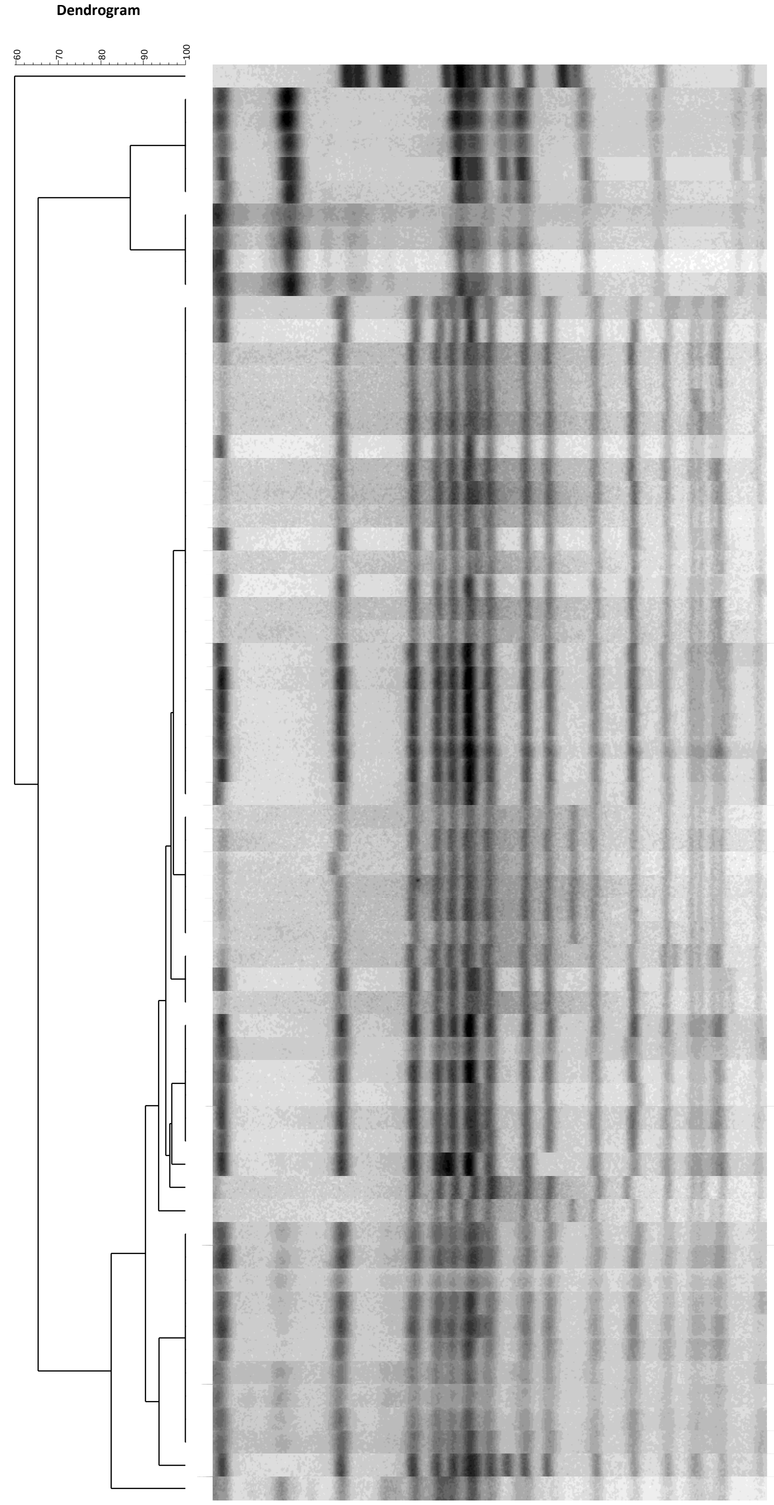

Key	Serotype	Genotype
3CT28	Corvallis	C-1
1CT52	Enteritidis	E-1
1CT29	Enteritidis	E-1
1CT28	Enteritidis	E-1
1CT19	Enteritidis	E-1
1CT147	Enteritidis	E-1
1CT58	Enteritidis	E-2
1CT116	Enteritidis	E-2
1CT76/#1	Enteritidis	E-2
1CT106	Enteritidis	E-2
1CT149	Infantis	I-9
1CT189	Infantis	I-1
6CT9	Infantis	I-1
6CT7	Infantis	I-1
6CT2	Infantis	I-1
6CT12	Infantis	I-1
1CT180	Infantis	I-1
6CT10	Infantis	I-1
4CT15	Infantis	I-1
3CT7	Infantis	I-1
1CT183	Infantis	I-1
5CT6	Infantis	I-1
1CT182	Infantis	I-1
6CT6	Infantis	I-1
3CT19	Infantis	I-1
2CT99	Infantis	I-1
2CT121	Infantis	I-1
2CT86	Infantis	I-1
2CT84	Infantis	I-1
2CT81	Infantis	I-1
1CT214	Infantis	I-1
1CT210	Infantis	I-1
3CT8	Infantis	I-2
3CT20	Infantis	I-2
2CT123	Infantis	I-2
3CT31	Infantis	I-2
3CT25	Infantis	I-2
3CT16	Infantis	I-2
6CT11	Infantis	I-12
1CT193	Infantis	I-3
3CT29	Infantis	I-3
2CT115	Infantis	I-4
1CT74	Infantis	I-4
1CT196	Infantis	I-4
1CT192	Infantis	I-4
2CT117	Infantis	I-4
1CT198	Infantis	I-4
2CT109	Infantis	I-5
4CT16	Infantis	I-6
3CT24	Infantis	I-7
1CT117	Infantis	I-8
1CT128	Infantis	I-8
1CT158	Infantis	I-8
1CT102	Infantis	I-8
1CT118	Infantis	I-8
1CT163	Infantis	I-8
1CT111	Infantis	I-8
1CT162	Infantis	I-8
1CT142	Infantis	I-8
1CT107	Infantis	I-8
1CT212	Infantis	I-10
1CT157	Infantis	I-11

Table A. Distribution of the minimal inhibitory concentration values for the 62 *Salmonella* isolates collected from the positive broiler batches

	Number of Salmonella isolates with minimal inhibitory concentrations (μg/μl)																
Antibiotic	0,02	0,03	0,06	0,12	0,25	0,5	1	2	4	8	16	32	64	128	256	512	1024
Sulfamethoxazole													2	2	3	1	54
Gentamicin					12	8		1	15	23	3						
Ciprofloxacin	5	2	3	20	26	6											
Ampicillin						4	9	6	1			42					
Cefotaxime			8	8	1	1	1		42			1					
Ceftazidime					11	5	6	36	4								
Tetracycline							5	4	2	1	1	48	1				
Streptomycin								3	5	2	3	31	15	3			
Trimethropim						11	1			2	1	47					
Chloramphenicol								13	6	2	1	1	39				
Colistin								52	10								
Florfenicol								14	4	2		1	41				
Kanamycin									30				2	30			
Nalidixic acid									7	1	1		53				

Full vertical lines indicate epidemiological break points for resistance described by European Committee on Antimicrobial Susceptibility Testing (EUCAST, 2015). Clinical break points for resistance described by the Clinical and Laboratory Standards Institute (CLSI, 2014) were used for Kanamycin and Sulfamethoxazole.
